# The key energy scales of Gd-based metallofullerene determined by resonant inelastic x-ray scattering spectroscopy

**DOI:** 10.1038/s41598-017-08685-5

**Published:** 2017-08-15

**Authors:** Yu-Cheng Shao, L. Andrew Wray, Shih-Wen Huang, Yi-Sheng Liu, Wang Song, Shangfeng Yang, Yi-De Chuang, Jinghua Guo, Way-Faung Pong

**Affiliations:** 1 Department of Physics, Tamkang University, Tamsui, 251 Taiwan; 2 Department of Physics, New York University, New York, New York 10003 USA; 3 MAX IV Laboratory, Lund University, P.O. Box 118, 22100 Lund, Sweden; 4 Advanced Light Source, Lawrence Berkeley National Laboratory, Berkeley, California 94720 USA; 5 Hefei National Laboratory for Physical Sciences at Microscale, CAS Key Laboratory of Materials for Energy Conversion, Department of Materials Science and Engineering, Synergetic Innovation Center of Quantum Information and Quantum Physics, University of Science and Technology of China, Hefei, 230026 China; 6 Department of Chemistry and Biochemistry, University of California, Santa Cruz, CA 95064 USA

## Abstract

Endohedral metallofullerenes, formed by encaging Gd inside fullerenes like C_80_, can exhibit enhanced proton relaxitivities compared with other Gd-chelates, making them the promising contrast agents for magnetic resonance imaging (MRI). However, the underlying key energy scales of Gd_*x*_Sc_3−*x*_N@C_80_ (*x*  =  1–3) remain unclear. Here, we carry out resonant inelastic x-ray scattering (RIXS) experiments on Gd_*x*_Sc_3−*x*_N@C_80_ at Gd *N*_4,5_-edges to directly study the electronic structure and spin flip excitations of Gd 4*f* electrons. Compared with reference Gd_2_O_3_ and contrast agent Gadodiamide, the features in the RIXS spectra of all metallofullerenes exhibit broader spectral lineshape and noticeable energy shift. Using atomic multiplet calculations, we have estimated the key energy scales such as the inter-site spin exchange field, intra-atomic 4*f*–4*f* Coulomb interactions, and spin-orbit coupling. The implications of these parameters to the 4*f* states of encapsulated Gd atoms are discussed.

## Introduction

When carbon atoms form three-dimensional structures like buckyballs (Buckminsterfullerene, a family of fullerene), the interior space can be used to store various types of atoms and molecules. The resulting compounds, known as endohedral fullerenes, have been proposed for various applications such as organic photovoltaic (OPVs) devices^1–3^, antimicrobial activity^4^, … etc. Among the encaged atomic species, lanthanides in particular Gd, have recently received attentions from medical community. Because of the large paramagnetic spin moment from 4*f* electrons of Gd^3+^ (S = 7/2), Gd-based compounds are extensively used as the contrast agents (CAs) in magnetic resonance imaging (MRI). However, the toxicity of Gd^3+^ ion to the human body requires it to be locked inside the chelates to be used as the contrast agents, which leads to significantly reduced proton relaxitivities *r*_1_ (longitudinal) and *r*_2_ (transverse) that are the key performance indices for MRI applications. Fullerenes, on the other hand, can serve as the natural cages for holding Gd^3+^ to prevent its leakage into the human body. When dressed with hydroxyl groups to enhance water solubility, the Gd-based endohedral metallofullerenols have been shown to have much higher proton relaxitivities compared with other Gd-chelates like Gd-DTPA^5–11^.

**Figure 1:**
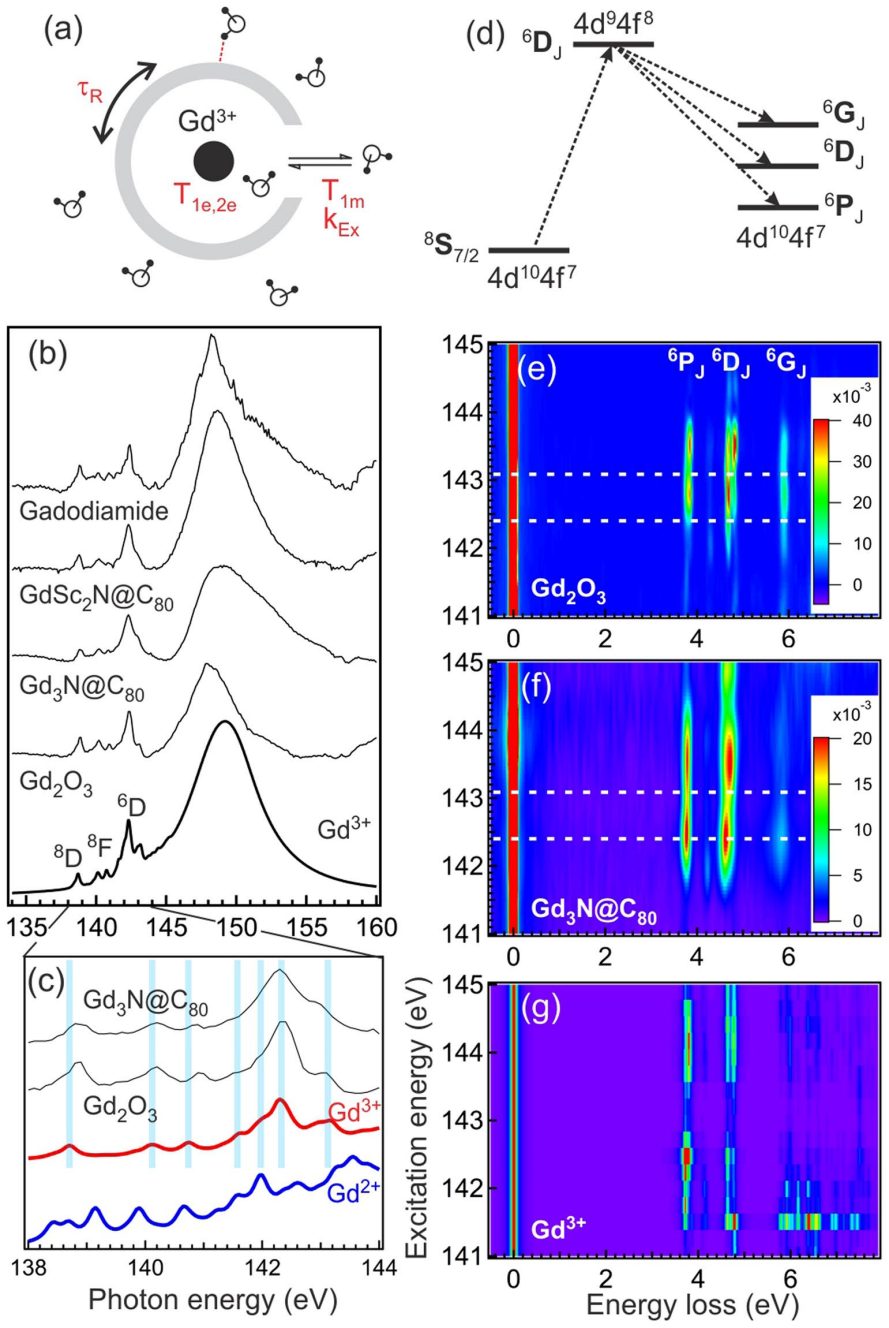
(**a**) Schematic illustration showing some key parameters affecting the MRI relaxivities: the electronic spin relaxation time of metal ions *T*_1*e*,2*e*_, the rotational correlation time *τ*_*R*_ and inner sphere water exchange reaction *T*_1*m*_ + *k*_*ex*_. (**b**) From top to bottom: experimental XAS spectra (thin lines) of Gadodiamide, Gd_1_Sc_2_N@C_80_, Gd_3_N@C_80_, and Gd_2_O_3_ compared with simulated XAS spectrum using single Gd^3+^ ion (thick line). Simulation parameters are listed in Table 1 (baseline). (**c**) Zoom-in pre-edge region of XAS spectra. The simulated spectra with Gd^3+^ and Gd^2+^ ion are denoted by thick red and blue lines, respectively. The blue vertical lines mark the main peak positions and are guides for eyes. (**d**) Schematic illustration of RIXS process producing the inelastic features with term symbols ^6^P_*J*_, ^6^D_*J*_, and ^6^G_*J*_ (in [3.5 eV, 8 eV] energy loss window). (**e**–**g**) RIXS maps of (**e**) Gd_2_O_3_, (**f**) Gd_3_N@C_80_, and (**g**) simulated Gd^3+^ single ion. The experimental RIXS maps in panels (e) and (f) are produced by interpolating the RIXS spectra recorded at 141, 141.5, 142, 142.4, 142.7, 143.1, 143.5, 144.5, and 145.5 eV excitation photon energies. The intensity of elastic peak (zero energy loss) is scaled to 1 with color bars shown on the right. White dashed lines in panels (e) and (f) denote the excitation photon energies used in Figs 2 and 3.

But unlike in Gd-chelates where water molecules (or H^+^) can come in contact with the central Gd^3+^ ion, the Gd^3+^ is well shielded by the carbon cage in metallofullerenes (EMFs). Therefore, relaxation mechanisms such as the inner sphere Gd^3+^-H^+^ spin interaction and the water exchange between inner and outer spheres are not applicable to EMFs. Instead, much of the discussions about the mechanisms behind their enhanced relaxitivities are on the rotational correlation time *τ*_*R*_ and the proton-fullerenol interaction (see Fig. 1(a) for the schematic illustration of some relaxation parameters)^11^. Although information such as the spin state and valence state of Gd in the fullerene and their interactions with carbon cage have been experimentally obtained^12^, important energy scales like spin-orbit coupling (SOC), intra-atomic 4*f*–4*f* Coulomb interactions, and inter-atomic spin exchange field J_*ex*_ in these Gd-based MRI contrast agents are at best calculated theoretically. These energy scales are significant parameters for determining the zero-field splitting (ZFS), an important factor that will influence the electronic relaxation of Gd spin (*T*_1*e*, 2*e*_) under the magnetic field^13, 14^. Since these key energy scales can affect the energetics of elementary excitations^15^, they can be determined by measuring the excitations associated with Gd using inelastically scattered x-rays under Gd resonance conditions.

In that regard, we have carried out the Gd *N*_4,5_-edges resonant inelastic x-ray scattering (RIXS) experiments on MRI contrast agent Gadodiamide, (Gd_*x*_Sc_3 − *x*_)N@C_80_ (x = 1–3), and Gd_2_ O_3_ (reference sample) to understand the effect of C_80_ fullerene on these energy scales. Our RIXS results with atomic multiplet calculations show that both SOC and Coulomb interactions between 4*f* multiplets of Gd^3+^ are reduced in the EMFs compared with Gadodiamide and Gd_2_O_3_. In addition, the 4*f*–4*f* Coulomb interactions play a more prominent role than J_*ex*_ in inducing these changes. Although the band gap of Gd EMFs are wider than the free Gd-cluster^16–20^, the *f*–*f* excitations created in the RIXS process are more renormalized in energy and shorter lived, implying the higher degree of orbital hybridization around Gd^3+^ ions in the EMFs.

## Results

### Gd *N*_4,5_-edge XAS spectra

The x-ray absorption (XAS) spectra of these samples around Gd *N*_4,5_-edges are shown as thin lines in Fig. 1(b). The simulated Gd^3+^ single ion XAS spectrum using parameters listed in the Experiment Method section is shown as a thick line. A strong resonance, known as the giant resonance, shows up around 148 eV in all spectra. This giant resonance originates from the dipole transitions from ground state to ^8^P_*J*_ final states that are broadened by autoionization^21^. Below this giant resonance, between 138 eV and 145 eV, there are several sharp features corresponding to the discrete Gd 4*d* → 4*f* transitions to ^8^D_*J*_, ^6^D_*J*_, and the much weaker ^8^F_*J*_ final states^22–24^.

The energies of these sharp features depend strongly on the valence state of Gd. In Fig. 1(c), we zoom in the [138 eV, 144 eV] energy window and compare the selected experimental XAS spectra with simulations using single Gd^3+^ (thick red line) and Gd^2+^ (thick blue line) ions. A good correspondence, highlighted by the blue vertical lines, can be seen between the peaks in Gd_2_O_3_, Gd_3_N@C_80_, and simulated Gd^3+^ XAS spectra. On the other hand, there is a gross discrepancy with simulated Gd^2+^ XAS spectrum where more peaks are predicted at different energies. The agreement implies that like in Gd_2_O_3_, the encaged Gd in EMFs has 3+ valence state (^8^S_7/2_ ground state), irrespective to the number of encaged Gd ions. This finding is consistent with previous XAS and EELS results^12, 25, 26^.

### Gd *N*_4,5_-edge RIXS spectra

Although the lineshape of giant resonance of these samples are slightly different, presumably reflecting slight Fano interference^27, 28^ and different degrees of orbital hybridization between Gd and the surrounding atoms, understanding the meaning of such difference would require a detailed model of how autoionization broadens the ^8^P_*J*_ multiplets. Furthermore, the short core hole lifetime (or large energy broadening) in XAS smears out fine spectral features that can reflect the influence of other low energy scale interactions. Thus in this case, the XAS spectra in Fig. 1(b) only provide the Gd valence state information. On the other hand, information about the local energetics like intra-atomic 4*f*–4*f* Coulomb interactions, inter-atomic spin exchange field J_*ex*_, and spin-orbit coupling (SOC) around the interacting Gd sites can be determined from RIXS spectra because the energies of various *f*–*f* excitations created in the |4*d* ^10^ 4*f* ^7^ >→|4*d* ^9^ 4*f* ^8^ >→|4*d* ^10^ 4*f* ^7^> RIXS process will depend strongly on these parameters (see Fig. 1(d) for the schematic illustration of RIXS process). In Fig. 1(e) and (f), we show the interpolated RIXS maps of Gd_2_O_3_ (Fig. 1(e)) and Gd_3_N@C_80_ (Fig. 1(f)) using the RIXS spectra recorded at excitation photon energies spanning over 141 eV to 145 eV. In these RIXS maps, the intensity of elastic peak (zero energy loss) is normalized to 1 to highlight the weak inelastic features in the energy loss range between 3.5 eV and 6 eV. The calculated Gd^3+^ RIXS map is also shown in Fig. 1(g).

**Figure 2:**
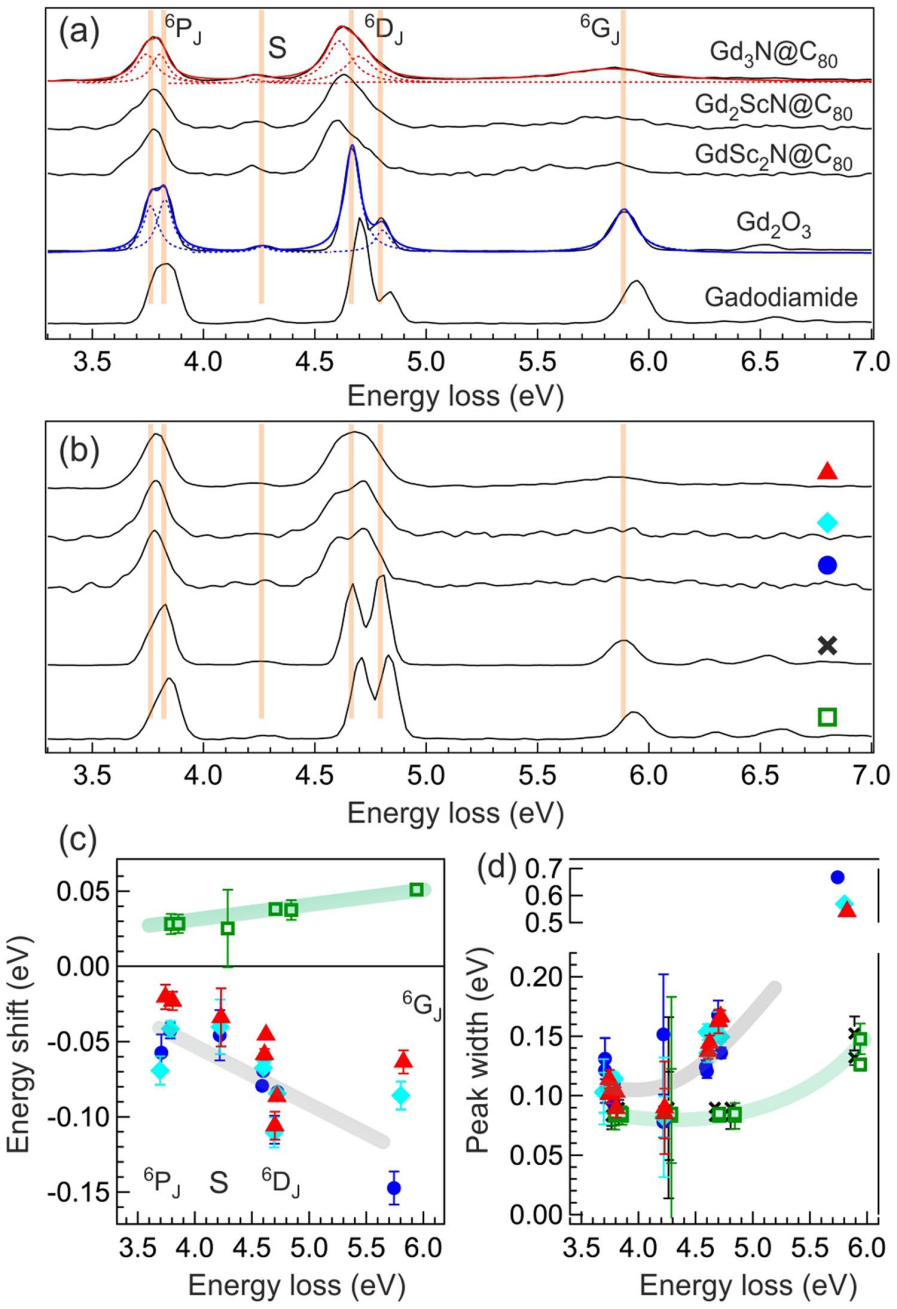
The RIXS spectra of (from top to bottom) Gd_3_N@C_80_, Gd_2_Sc_1_N@C_80_, Gd_1_Sc_2_N@C_80_, Gd_2_O_3_, and Gadodiamide recorded at excitation energy of (**a**) 142.4 eV (at ^6^D_9/2_ in XAS) and (**b**) 143.1 eV (at ^6^D_7/2_ in XAS). The RIXS spectra are normalized to the area of ^6^D_*J*_ feature and offset vertically for clarity. The orange vertical lines mark the main peaks in Gd_2_O_3_ and are guides for eyes. The red and blue curves in panel (a) show the fitted Lorentzian functions for Gd_3_N@C_80_ and Gd_2_O_3_, respectively. (**c**) Relative peak positions (relative to the peaks in Gd_2_O_3_) plotted against the energies of the peaks in Gd_2_O_3_ and (**d**) full width at half maximum (FWHM) from Lorentzian fitting of RIXS spectra in panels (a) and (b). Different symbols are used to denote the results for Gd_3_N@C_80_ (red filled triangle), Gd_2_Sc_1_N@C_80_ (cyan filled diamond), Gd_1_Sc_2_N@C_80_ (blue filled circle), Gd_2_O_3_ (black cross), and Gadodiamide (green open square). The green and gray lines are guides for eyes.

At first glance, both experimental RIXS maps exhibit similar inelastic features that show strong resonance enhancement between 142 eV and 144 eV excitation photon energies. These features are faithfully reproduced in the Gd^3+^ RIXS simulation. They have been identified as the *f*-*f* excitations created in the transitions from octet ^8^S_7/2_ ground state to ^6^D_*J*_ intermediate states, and then to the sextet (^6^P_*J*_, ^6^D_*J*_, ^6^G_*J*_) final states (spin flip excitations; see Fig. 1(d))^22–24^. With improved energy resolution over previous RIXS measurements, some finer spectral structures can be revealed even in these RIXS maps: the splitting of ^6^P_*J*_ and ^6^D_*J*_ features, and the weak satellite feature S in between them (see labels in Fig. 2(a)). One notices that in addition to the lower intensity by about a factor of 2, the RIXS features in Gd_3_N@C_80_ are not as well resolved as those in Gd_2_O_3_. In particular, the ^6^G_*J*_ manifold is considerably weaker and broader in Gd_3_N@C_80_.

To further analyze the data, we show the RIXS spectra from these samples taken at two excitation photon energies, 142.4 eV (Fig. 2(a)) and 143.1 eV (Fig. 2(b)), as denoted by the white dotted lines in Fig. 1(e) and (f). In these figures, the RIXS spectra are normalized using the area of ^6^D_*J*_ feature and offset vertically for clarity (peak area determined from the Lorentzian fitting; see later discussion). One can see that relative to the main peaks in Gd_2_O_3_ RIXS spectra that are highlighted by the orange lines, the corresponding peaks in Gadodiamide RIXS spectra are shifted towards higher energy loss, whereas they are shifted towards lower energy loss in the EMF RIXS spectra. In addition, they display different width behaviors: the main peaks in the EMF RIXS spectra are much broader than those in Gd_2_O_3_ and Gadodiamide, irrespective to the excitation photon energies or the number of encaged Gd^3+^ ions.

To quantify these differences, we use five Lorentzian functions to fit the ^6^P_*J*_, ^6^D_*J*_, and ^6^G_*J*_ features (two for ^6^P_*J*_ and ^6^D_*J*_, and one for ^6^G_*J*_) and one Lorentzian function to fit the satellite feature S. The red and blue dashed lines in Fig. 2(a) show the representative fitting results for Gd_3_N@C_80_ and Gd_2_O_3_, respectively. In the fitting routine, the widths (full width at half maximum, FWHM) of these Lorentzian functions are constrained to be equal or larger than that of the corresponding elastic peaks, which vary from 75 meV to 90 meV at these excitation photon energies. We notice that the excess intensity in the tails of the Lorentzian fit curves for Gd_2_O_3_ excitations is an indication that these measurements are partially limited by the ~80 meV experimental resolution. The resulting peak positions relative to the ones in Gd_2_O_3_ and the widths are summarized in Fig. 2(c) and (d), respectively. The fitting results from two excitation photon energies are combined in the same figures.

From Fig. 2(c), one can see that the relative changes in the peak energy positions are not constant, but depend on the energy loss: the higher the energy loss is, the large the energy shift becomes. They exhibit a roughly linear scaling behavior except for the satellite feature that seems to have a smaller energy shift relative to other RIXS features. For EMFs, the ^6^G_*J*_ features in the RIXS spectra are so weak and broad that fitting their energy positions is challenging and can sometimes be unreliable. Thus for spectra that simply do not yield reliable peak positions, the results are not included in Fig. 2(c) and (d). From the width behaviors in Fig. 2(d), we see that the peaks in the EMF RIXS spectra are about a factor of ~1.5 for ^6^P_*J*_, ~2 for ^6^D_*J*_, and >5 for ^6^G_*J*_ broader than those in either Gd_2_O_3_ or Gadodiamide. As in between the EMFs, with limited statistics in the experimental RIXS spectra and the scattered Loretzian fitting results, we do not see clear distinction in their peak positions and widths.

### Atomic multiplet calculations for Gd^3+^

**Figure 3:**
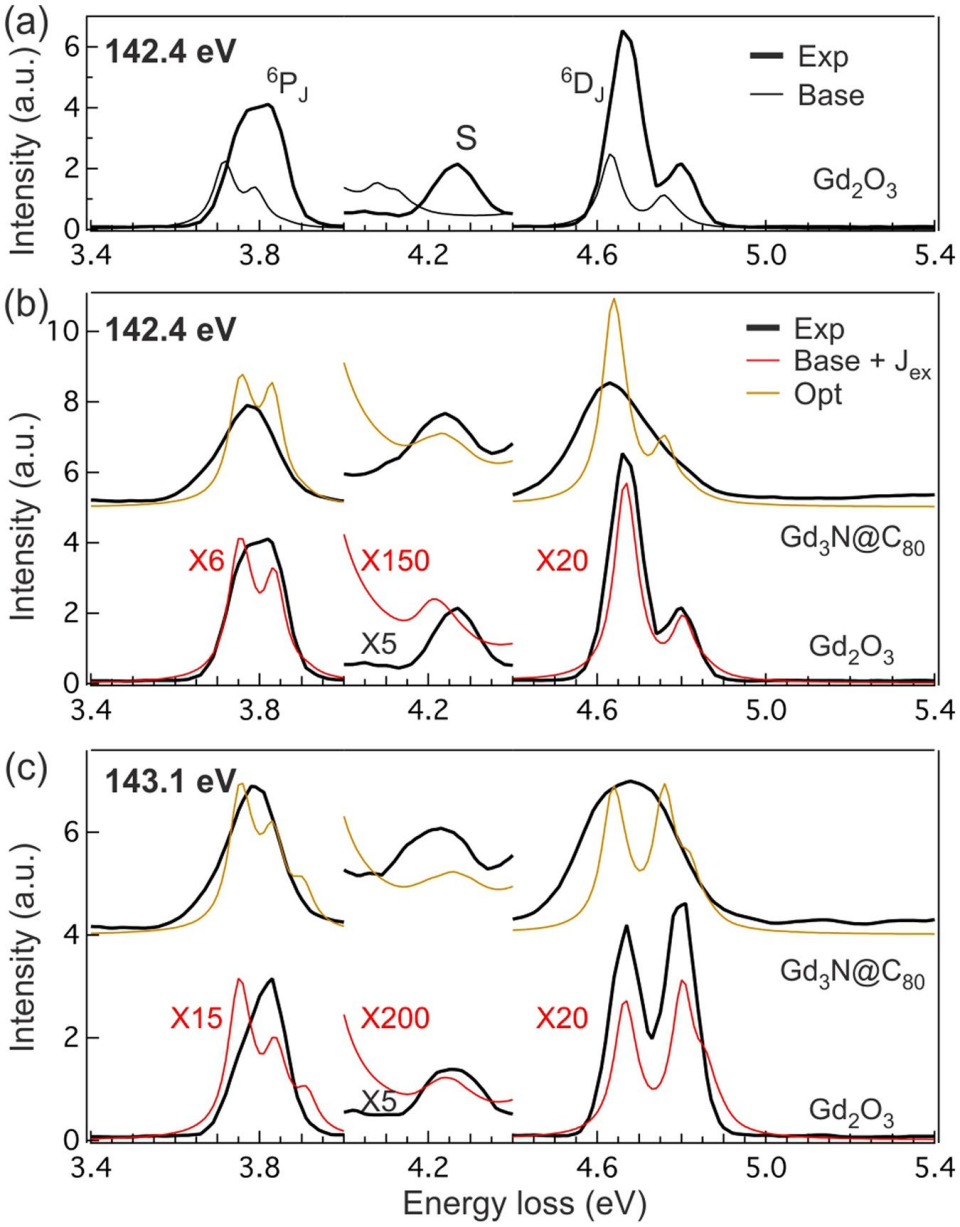
(**a**) Comparison of Gd_2_O_3_ (thick black line) and simulated Gd^3+^ (baseline; thin black line) RIXS spectra at 142.4 eV excitation photon energy. The simulation parameters for baseline spectrum are listed in Table 1. (**b**,**c**) Comparison of Gd_3_N@C_80_ (thick black line) and simulated RIXS spectra (thin yellow line) with optimized energy parameters (1% reduction in 4*f*–4*f* Coulomb interactions, 10% reduction in SOC, and J_*ex*_ = 40 meV; parameters are listed in Table 1) at excitation photon energy of (**b**) 142.4 eV and (**c**) 143.1 eV. The experimental Gd_2_O_3_ RIXS spectra (thick black line) and baseline simulations plus J_*ex*_ = 30 meV (thin red line) are also shown in the bottom part of the same figures. For comparison, spectra are scaled differently in different energy windows. For 142.4 (143.1) eV excitation energy, the simulation is scaled up by a factor of 6 (15) in [3.4 eV, 4.0 eV] window; in [4.0 eV, 4.4 eV] window, the simulation and experiment are scaled up by a factor of 150 (200) and 5 (5), respectively; in [4.4 eV, 5.4 eV] window, the simulation is scaled up by a factor of 20 (20). The scaling factors (red for simulations and black for experiments) are also listed in the figures.

To understand the meaning of distinctive behaviors of peak positions in these RIXS spectra, we examine the dependence of atomic multiplet simulations on different physical parameters. In Fig. 3(a), we compare the Gd_2_O_3_ RIXS spectrum (thick black line) with single ion Gd^3+^ simulation (thin black line) using the 4 *f*-4 *f* Coulomb interactions and SOC described in the Experimental Methods section (baseline spectrum with J_*ex*_ = 0 meV; parameters also listed in Table 1). For comparison, the spectra are scaled differently in three different energy windows (see figure caption). We notice that the baseline simulation does not completely reproduce the observed Gd_2_O_3_ RIXS lineshape: the intensities of ^6^P_7/2_ and ^6^P_5/2_ are comparable in Gd_2_O_3_ RIXS spectrum, whereas they are very different in the baseline simulation; the simulation produces much stronger ^6^P_*J*_ features relative to ^6^D_*J*_ (note the different scaling factors for simulation); the main peaks in the simulation are shifted towards lower energy loss, with satellite feature exhibiting larger shift. We attribute these intensity and lineshape discrepancies to the elastic line normalization procedure and the limited single ion model used in the simulations. Despite such caveat, the simulation reproduces the main features highlighted by the orange lines in Fig. 2(a) and (b).

**Table 1:** Parameters used in atomic multiplet calculations (in units of eV).

Baseline simulation without core hole, J_*ex*_ = 0 meV									
4 *f*-4 *f* Slater-Condon parameters									
F^2^	F^4^	F^6^	4*f* SOC						
11.2668	7.071	5.087	0.1815						
Baseline simulation with core hole, J_*ex*_ = 0 meV									
4*f*–4*f* Slater-Condon parameters			4*d*–4*f* Slater-Condon parameters						
F^2^	F^4^	F^6^	G^1^	G^3^	G^5^	F^2^	F^4^	4*f* SOC	4*d* SOC
12.5202	7.8629	5.6589	12.5857	7.9071	5.5926	10.6692	6.8208	0.1851	2.409
Simulation for EMF without core hole									
4*f*–4*f* Slater-Condon parameters									
F^2^	F^4^	F^6^	4*f* SOC	J_*ex*_					
11.1541	7.0003	5.0361	0.1635	0.04					

**Figure 4:**
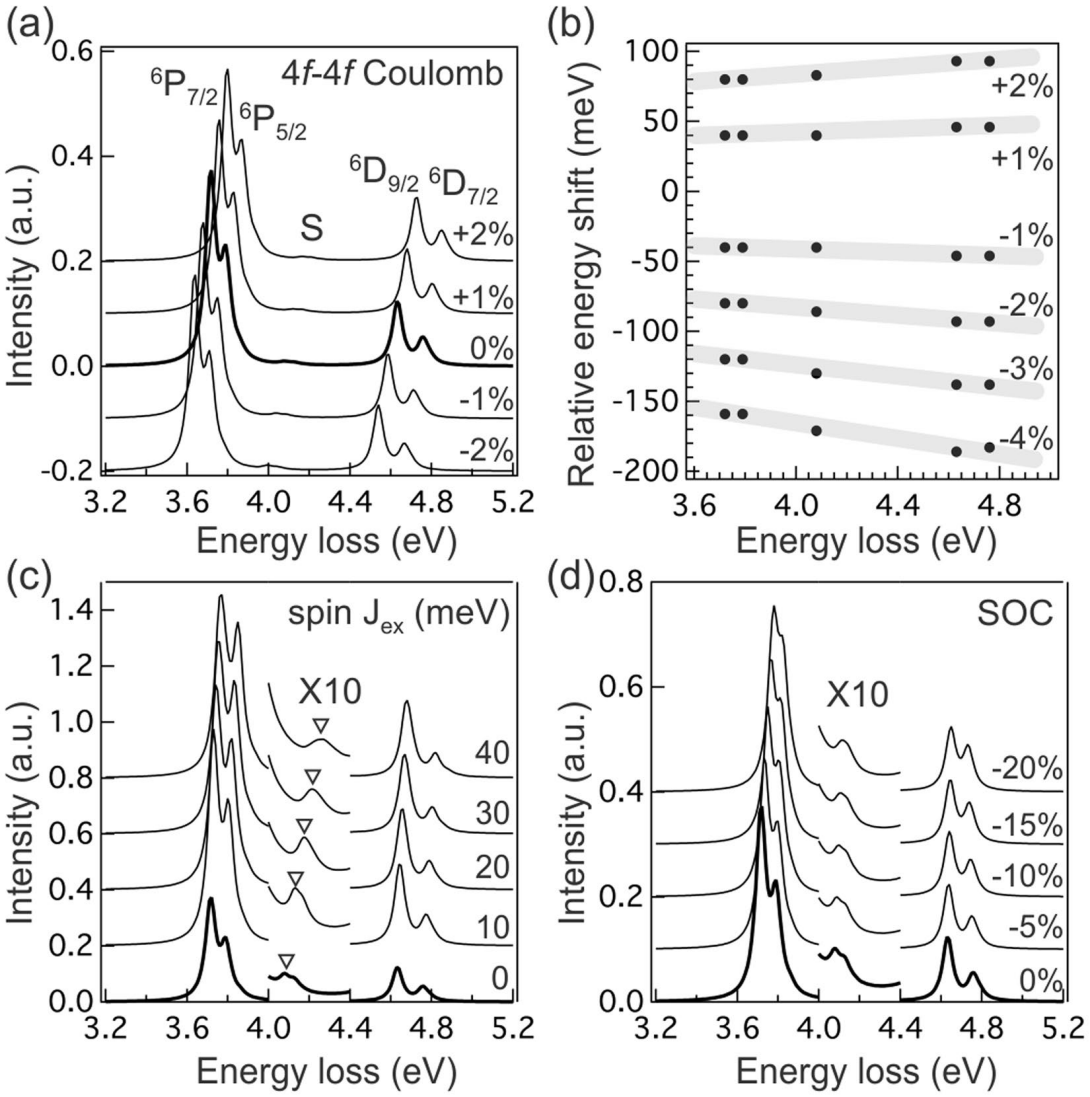
(**a**) Simulated RIXS spectra with varying 4*f*–4*f* Coulomb interactions. The amount of variation relative to the baseline parameters is listed in the figure. (**b**) Relative peak positions plotted against the ones with 0% variation (thick line) in 4*f*–4*f* Coulomb interactions. The highlighted straight lines are guides for eyes. The percentage of variation in 4*f*–4*f* Coulomb interactions is listed in the figure. (**c**) Simulated RIXS spectra with varying spin exchange field J_*ex*_. The spectra in [4.0 eV, 4.4 eV] energy loss window are magnified by a factor 10 to show the shift of satellite feature S. The magnitude of J_*ex*_ is listed in the figure. The inverted triangles mark the peak position and are guides for eyes. (**d**) Simulated RIXS spectra with varying spin-orbit coupling (SOC). The spectra in [4.0 eV, 4.4 eV] energy loss window are magnified by a factor 10. The percentage of reduction in SOC is listed in the figure. Spectra in panels (a,c and d) are shifted vertically for clarity.

In Figs 4(a–d), we vary these key energy parameters relative to the baseline simulation (thick line in these figures) to explore the energy shift of main RIXS features. By setting the excitation photon energy to 142.4 eV and varying the key energy parameters, several salient trends can be deduced. When reducing (increasing) the Coulomb interactions between 4*f* electrons (4*f*-4*f* Slater integrals), the peaks are shifted towards lower (higher) energy loss without introducing new features in the spectra (see Fig. 4(a)). As shown in Fig. 4(b) where we plot the energy positions of ^6^P_*J*_, S, and ^6^D_*J*_ features relative to the ones in the baseline spectrum, the relative energy shift scales roughly linearly with fractional change in 4 *f*-4 *f* Coulomb interactions [the slope of highlighted lines in the figure changes almost linearly with Coulomb interactions]. Thus the observed peak energy shift in Fig. 2(c) can be attributed mainly to different 4 *f*-4 *f* Coulomb interactions experienced by Gd^3+^ in the EMFs (decreased Coulomb interactions) and Gadodiamides (increased Coulomb interactions) relative to Gd_2_O_3_. As for EMFs, we see similar magnitude of energy shift, irrespective to how many Gd^3+^ ions are encaged in the fullerene. This finding suggests that the C_80_ cage offers a similar hybridization environment for Gd^3+^ ions.

Increasing the strength of spin exchange field J_*ex*_ shifts the peaks to higher energy loss, and the amount of energy shift for the main ^6^P_7/2_ and ^6^D_9/2_ feature is comparable (see Fig. 4(c)); however, we notice that the magnitude of energy shift with J_*ex*_ is smaller than that of the 4 *f*-4 *f* Coulomb interactions. Increasing J_*ex*_ will also increase the energy separation between ^6^P_7/2_ and ^6^P_5/2_, ^6^D_9/2_ and ^6^D_7/2_ manifolds and slightly enhance the intensities of even weaker excitations, resulting in broader spectral profiles in general. Intriguingly, the biggest effect of changing J_*ex*_ is on the satellite S: the energy shift for S marked by the inverted triangles in the figure is much larger than other features in the spectra. This is related to the nature of this satellite, which contains excitations with ^6^I_7/2,9/2,11/2_ symmetry. Because of the large orbital angular momentum L = 6, spin-orbit coupling results in greatly reduced spin moment for these excitations. The significantly reduced spin polarizability causes the ^6^I_*J*_ satellite feature to appear at higher energy loss relative to other smaller L features when the effects of increased J_*ex*_ on the surrounding environment are considered. Therefore, the energy position of satellite S can be used to determine the spin exchange J_*ex*_ in these materials. Reducing the SOC has almost no effect on the energies of the satellite S or ^6^D_*J*_ features, but the ^6^P_*J*_ and ^6^G_*J*_ features are shifted towards higher energy loss (see Fig. 4(d); the ^6^G_*J*_ features are not shown in the figure). In addition to reducing the energy separation between ^6^P_7/2_ and ^6^P_5/2_, and ^6^D_9/2_ and ^6^D_7/2_, reducing the SOC will also enhance the intensities of features with smaller J.

## Discussion

The aforementioned trends with respect to varying the effective atomic energy parameters are independent of excitation photon energy. Based on the simulations in Fig. 4(a–d), we overlay the Gd_3_N@C_80_ RIXS spectra (thick black line, top) with simulated one (thin yellow line) using 1% reduced 4 *f*-4 *f* Coulomb interactions, 10% reduced SOC, and J_*ex*_ = 40 meV in Fig. 3(b) (142.4 eV) and 3(c) (143.1 eV), respectively. The simulation parameters are also summarized in Table 1. In the same figures, we also show the Gd_2_O_3_ RIXS spectra (thick black line, bottom) with baseline simulation plus J_*ex*_ = 30 meV (thin red line). The choice of J_*ex*_ values is based on the position of satellite S relative to other excitations, and not on the shift with respect to sample chemistry or temperature, which would be more accurate. Nonetheless, the methods presented here provide a framework for comparing between different chemistries based on RIXS spectra. The simulated spectra show the correct energy shift in the Gd_3_N@C_80_ relative to Gd_2_O_3_, as well as for the satellite feature relative to other RIXS features. The J_*ex*_ and 4 *f*-4 *f* Coulomb interaction values used in the simulations are similar to recent LDA + U calculations^16^, providing the experimental confirmation of these important energy parameters.

Although the atomic multiplet calculations with 60 meV Lorentzian broadening can reproduce the energies of main features, the much broader widths seen in the experimental RIXS spectra in Figs 2(d) and 3 suggest other effects besides the instrument energy resolution. Broadening of inelastic features has been seen in the RIXS spectra of transition metal oxides and dilute magnetic semiconductors, and has been attributed to the shorter final state lifetime from the rapid decay of excitations (*d*-*d* excitations in these literatures) to the electron-hole pairs in the host valence and conduction bands through ligand band hybridization^29, 30^. This final state lifetime effect would have the largest influence on states with highest spin or orbital angular momentum, which are the ^6^G_*J*_ states in this study. In the EMFs, the possibility of weak charge-transfer between Gd^3+^ 4* f* states and carbon cage was suggested by x-ray magnetic circular dichroism (XMCD)^31^. Such a charge-transfer scenario, implying certain covalency for Gd^3+^ 4* f* states, would be in line with our RIXS findings. The more delocalized Gd^3+^ 4*f* states may also play a role in the enhanced relaxitivities in the EMFs, as recent studies on Gd_2_O_3_ nanoparticles showed enhanced relaxitivities when particle size approaches to 3 nm that Gd 4* f* wave functions become comparable to the particle size^32, 33^.

In conclusion, we have carried out XAS and RIXS measurements on endohedral metallofullerenes Gd_*x*_Sc_3 − *x*_N@C_80_ (x = 1–3), Gadodiamide, and reference Gd_2_O_3_ powders to study the energy parameters in these materials. XAS results show that the valence state of Gd is 3+ in these materials, consistent with previous XAS and EELS results. RIXS measurements at 142.4 eV (at ^6^D_7/2_ final state in XAS) and 143.1 eV (at ^6^D_9/2_ final state in XAS) show weak *f*- *f* excitations that are energy shifted with respect to the ones in Gd_2_O_3_: the peaks in EMFs are consistently shifted towards lower energy loss whereas they are shifted towards higher energy loss in Gadodiamide. Using atomic multiplet calculations, we show that such shifts in peak positions are related to different key energetic parameters of the Gd electrons like intra-atomic 4 *f*-4 *f* Coulomb interactions, inter-atomic spin exchange field J_*ex*_, and spin-orbit coupling in these materials. We have estimated that relative to Gd_2_O_3_, the 1% reduction in 4 *f*-4 *f* Coulomb interactions, 10% reduction in spin-orbit coupling, and J_*ex*_ = 40 meV produce the simulated spectrum that agrees better with the experimental EMFs RIXS spectra. The large peak widths in the EMF RIXS spectra, which are a factor of ~1.5 to >5 larger than those in Gd_2_O_3_ and Gadodiamide, we argue reflect the shorter spin flip excitation lifetimes. Such shorter lifetimes, in conjunction with reduced 4*f*–4*f* Coulomb interactions, can come from finite orbital hybridization between Gd 4* f* states and carbon on the cage. This finding suggests that even though EMFs are small band gap insulators, the Gd^3+^ ions inside the cage are less ionic than those in the Gd-chelates like Gadodiamide that exhibits enhanced 4 *f*-4 *f* Coulomb interactions. In addition, the partial delocalization of Gd 4* f* electrons may also play a role in the enhanced relaxitivities in EMFs, as suggested by the recent studies on 3 nm Gd_2_O_3_ nanoparticles.

## Experimental Methods

### Samples

In this study, some powder samples were purchased from the commercial sources: Gd_2_O_3_ (40 nm particle size) and Gadodiamide came from Sigma-Aldrich, and Gd_3_N@C_80_ powder with 95% purity came from SES Research. These commercial powders were packed and pressed onto the indium foils for handling in the experiments. Gd_1_Sc_2_N@C_80_ and Gd_2_Sc_1_N@C_80_ were fabricated using Kr ä tschmer-Huffman arc burning method^34^. With extremely limited quantity, the synthesized powders were first dissolved in toluene and then dropped onto the gold foils. After evaporating the toluene, a thin layer of Gd EMF powder was left on the surface of gold foil. All foils were attached to the sample holders using carbon tape and loaded into the experimental chamber from airside through the loadlock chamber.

### XAS and RIXS measurements

The experiments were carried out at BL4.0.3 (MERLIN) and BL8.0.1 at the Advanced Light Source (ALS), Lawrence Berkeley National Laboratory (LBNL), using the MERIXS endstation. All x-ray absorption (XAS) and resonant inelastic x-ray scattering (RIXS) measurements were performed at room temperature in the UHV environment (vacuum better than 5 × 10^−10^ torr). The XAS spectra shown in this paper were recorded using the total electron yield (TEY) mode of detection (sample-to-ground drain current). The spectra were normalized by the photocurrent from an upstream gold mesh in the beamline. The RIXS spectra were recorded using the high-resolution spectrometer placed at 90° emission angle relative to the incident photon beam^35^. With photon polarization in the horizontal scattering plane (*π*-polarization), the intensity of elastic peak was significantly suppressed to reveal the low energy excitations. The energy resolution (beamline and spectrometer combined) determined from the full-width at half maximum (FWHM) of the elastic peak was between 75 meV and 90 meV.

### Atomic multiplet calculations

The scattering process was modeled using the Kramers-Heisenberg equation with an atomic multiplet basis, as in ref. 28. The F and G terms are defined in ref. 36, and describe the decomposition of Coulomb interactions by Legendre polynomial order. More details can be found in refs 37 and 38. Simulations were performed by evaluating transitions between the diagonalized ground states and core hole states of the multiplet model, in the dipole approximation. Full diagonalization of the Hamiltonian was performed using LAPACK drivers^39^. The Hartree-Fock parameters used in the calculations were renormalized (parameters listed here are for the baseline spectrum, which are chosen to simulate the Gd_2_O_3_ RIXS spectrum with 142.4 eV excitation photon energy after adding an exchange field J_*ex*_ ~30 meV; see previous discussion): relative to the Hartree-Fock values, the 4 *f*-4 *f* Slater-Condon parameters (F^2^, F^4^, and F^6^) were reduced to 86% and 78% with and without a core hole, respectively; the 4* f* spin-orbit coupling was slightly reduced to 92%; with a core hole, the 4 *d*-4 *f* Slater-Condon parameters (F^2^, F^4^, G^1^, G^3^, and G^5^) were reduced to 65%; the 4 *d* spin-orbit coupling was increased to 110% as a slight increase can be necessary for this parameter in the context of very shallow core holes on a positive valence site^40^. These parameters in units of eV are also summarized in Table 1. Core hole lifetime was set to Γ = 0.3 eV for the leading edge states, and Γ = 4.5 eV for states above 146 eV, which are likely above the autoionization threshold^41, 42^. The simulated energy loss spectra were broadened by a Lorentzian function with 60 meV FWHM. In the discussion section, we also present the simulations with varying the aforementioned parameters to examine the evolution of different RIXS features. Changes relative to these baseline parameters are denoted in the figures.
